# Refractory *Scedosporium apiospermum* Keratitis Successfully Treated with Combination of Amphotericin B and Voriconazole

**DOI:** 10.1155/2013/413953

**Published:** 2013-02-20

**Authors:** Mohd-Tahir Fadzillah, Siti-Raihan Ishak, Mohtar Ibrahim

**Affiliations:** Department of Ophthalmology, School of Medical Sciences, Universiti Sains Malaysia, 16150 Kubang Kerian, Kelantan, Malaysia

## Abstract

*Aim*. To report a case of refractory fungal keratitis caused by *Scedosporium apiospermum*. *Methods*. Interventional case report. *Results*. A 47-year-old Malay housewife presented with left eye cornea ulcer as her first presentation of diabetes mellitus. There was no history of ocular trauma, contact lens used, or cornea foreign body. *Scedosporium apiospermum* was isolated from the cornea scrapping. Her cornea ulcer initially responded well to topical Amphotericin B within 3 days but subsequently worsened. Repeat cornea scrapping also yields *Scedosporium apiospermum*. This refractory keratitis was successfully treated with a combination of topical Amphotericin B and Voriconazole over 6 weeks. *Conclusion*. *Scedosporium apiospermum* keratitis is an opportunistic infection, which is difficult to treat despite tight control of diabetes mellitus and intensive antifungal treatment. The infection appeared to have very quick onset but needed long duration of treatment to completely heal. Surgical debridement always plays an important role as a therapeutic procedure as well as establishes the diagnosis through repeat scrapping.

## 1. Introduction 

Fungal keratitis remains as a challenge to ophthalmologists through out the world. The incidence of fungal keratitis is particularly higher in agricultural and developing countries [1]. The most common pathogens reported are *Fusarium* species and *Aspergillus* species [2].* Scedosporium* species commonly found in soil or decaying plant are previously thought to be opportunistic infection in immunocompromised individual only. Recently, *Scedosporium apiospermum* and its sexual form, *Pseudallescheria boydii* have been identified as emerging opportunistic pathogen responsible for mould infection in immunocompromised and occasionally immunocompetent patients [3] with no exception to keratitis. In fact, keratitis is the most common infection of *Scedosporium apiospermum* in immunocompetent patient. Here, we report the outcome of our first case of *Scedosporium* keratitis, successfully treated with combination of topical Amphotericin B and Voriconazole.

## 2. Case Report

**Figure 1:**
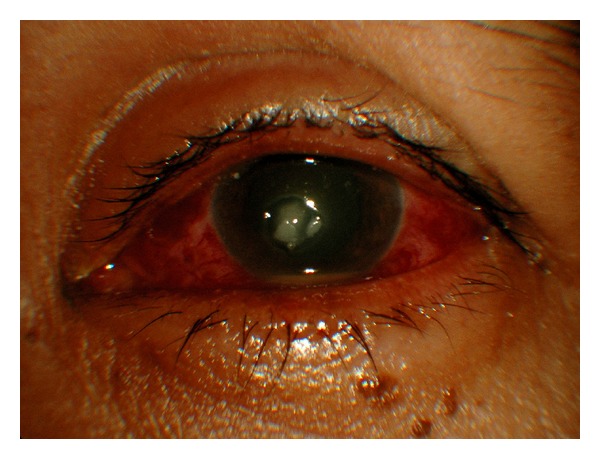
Left eye cornea ulcer with level of hypopyon at presentation.

A 47-year-old housewife presented with 2 days history of foreign body sensation and reduced vision over the left eye (Figure 1). There was no history of trauma. She has never used glasses or contact lens in the past as she claimed that her visual acuity on each eye was equally good. On examination, her visual acuity was 6/9 in the right eye and counting finger in the left eye. The conjunctiva of the left eye was injected. There was no foreign body. There was a stromal abscess at the centre of the cornea measuring 2.8 mm × 2.5 mm with overlying cornea epithelial defect of 2.0 mm size in diameter. The edge of the abscess was well defined. There was no endothelial plaque or satellite lesion to suggest fungal keratitis. The left eye anterior chamber filled with inflammatory cells and a level of hypopyon (0.8 mm). Cornea sensation on the left eye was slightly reduced compared to the right eye. The left eye posterior segment was hazy in view. Both eyes have similar intraocular pressure ranging from 14 mmHg to 16 mmHg. The right eye was entirely normal.

Topical Ceftazidime 1% and Gentamicin 0.3% eye drops were commenced after cornea scrapping. She was admitted the same day for intensive treatment of the left cornea ulcer. Later that day, fungal hyphae were seen on cornea sample under the microscope. Gram stain for bacteria was negative. She was started on eye drop of Amphotericin B 0.15% while Gentamicin eye drop was changed to Ciprofloxacin eye drop. On the day of admission, her random capillary blood sugar was found out to be 30.6 mmol/L. She is only known to suffer from hypertension for the past 4 years. Her blood sugar was subsequently well controlled with two types of oral hypoglycaemic agent and comanagement with dietician. The hypopyon in the left eye has completely disappeared after 3 days on treatment.

However, the next day, we noticed that the abscess started to have more obvious endothelial plaque. There was a new level of hypopyon on the following day. As the abscess progressively become thicker over a week, she underwent deep cornea debridement under local anaesthesia. Another cornea sample was sent for gram stain and cultures. No fungal element was seen this time and bacteria gram stain was also negative. The following day, the first cornea scraping was reported to isolate *Scedosporium apiospermum*. Oral Fluconazole 200 mg daily and hourly Fluconazole eye drop were initiated while the frequency of topical Ciprofloxacin eye drop was reduced. The oral Fluconazole was increased to twice a day on the next day as her left eye worsens. The left eye progressively worsens over the next five days despite combinations of systemic antifungal, two types of antifungal eye drop and tight control of capillary blood sugar (ranging from 4 to 6 mmol/L). At this stage, we stopped the Fluconazole and put her on daily dose of 200 mg oral Voriconazole and hourly Voriconazole 1% eye drop which was prepared extemporaneously. 

**Figure 2:**
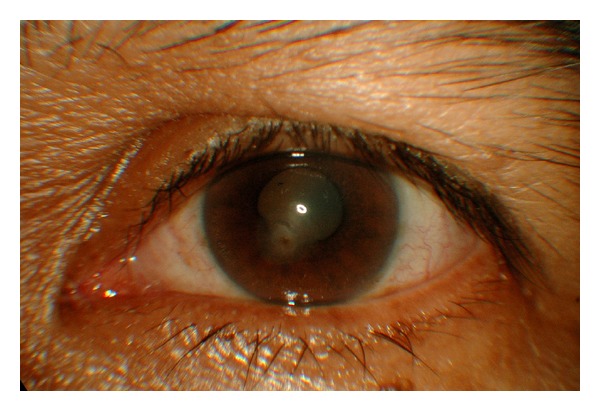
Left eye cornea ulcer healed following successful treatment.

The left eye showed slow improvement with the combination of Amphotericin B and Voriconazole. Two days later, the second scraping has isolated *Scedosporium apiospermum*. After a week, she was discharged against medical advice due to family problem. She remains compliant to her treatment and was self-admitted 4 days later. The left eye slowly improved where the hypopyon resolved after 5 weeks on the combination of Amphotericin B and Voriconazole. Oral Voriconazole was administered till 6 weeks. Topical Amphotericin B was used for 4 months while the topical Voriconazole was used for 8 months. She developed no adverse reaction throughout the treatment. Her best corrected vision upon completion of medication was 6/12. She remains asymptomatic with visual acuity of 6/9 in each eye on a year followup (Figure 2).

## 3. Discussion

Fungal keratitis caused by *Scedosporium apiospermum* is rare. More commonly responsible pathogens for fungal keratitis are *Fusarium* species and *Aspergillus* species. In agricultural or developing countries, being a farmer is one of the risk factors for this type of keratitis. Our case is unique where infection of *Scedosporium apiospermum* involved a housewife with no agricultural activity. With no history of trauma or contact lens usage, one possible explanation for the ulcer in this case is diabetic cornea erosion that subsequently infected by fungus from contaminated water. Nonhealing cornea ulcer may rarely become the first presentation of diabetes mellitus [4, 5]. It is even rare here as fungal keratitis caused by *Scedosporium apiospermum* is the first manifestation of diabetes in our patient.

Different modalities of treatment have been used for treating infection caused by this rare pathogen which is also known to have high level of antifungal resistance [3]. Despite having variable resistances, some researchers believe that different strains of *Scedosporium* have different virulence [6]; therefore, treatment might be different throughout the world. Furthermore, studies on susceptibility of different strains of *Scedosporium* on conventional antifungal and studies on different modalities of treatment were usually done in vitro. In vivo susceptibility of *Scedosporium* infection of cornea relies heavily on case reports because clinical trials are difficult to conduct. To our knowledge, up to date, there was no report on *Scedosporium apiospermum* keratitis successfully treated with combination of topical Amphotericin B and Voriconazole. Being the first one, treatments used in our first case of *Scedosporium* keratitis are solely based on clinical response and the literature reviewed.

In vitro and in vivo susceptibility of *Scedosporium apiospermum* to conventional antifungal could differ, especially to Amphotericin B. *Scedosporium apiospermum* keratitis successfully treated with Amphotericin B alone has been reported despite apparent in vitro resistance of the isolate to this drug and all other antifungal [7]. Walsh et al. found that some strains of *Scedosporium* species were susceptible to Amphotericin B [8]. Our case showed good response to Amphotericin B immediately but worsens subsequently. In vitro synergistic effect of Amphotericin B when combined with various azoles has been reported by Walsh et al. [8]. It is believed that greater antifungal activities were achieved when simultaneous exposure of the fungus to both antifungals resulted in increased permeability to the azole with increased inhibition of fungal ergosterol synthesis. In the study, the combination of Amphotericin B and Fluconazole displayed the greatest synergy. However, our case showed no improvement with this combination; therefore, we switched from Fluconazole to Voriconazole.

Nulens et al. reported that after 12 days on oral Voriconazole, the level of Voriconazole in the aqueous humor may achieve up to 53% of the level in plasma and exceeded the minimal inhibitory concentration (MIC) for the isolate by sevenfold [9]. In his case, both Itraconazole and Amphotericin B failed to treat this refractory infection. The patient had penetrating keratoplasty and oral Voriconazole was initiated following the surgery. Two loading doses of oral Voriconazole 6 mg/kg of body weight followed by 4 mg/kg twice a day for 3 months were used in his case. Our case is in agreement with 2 cases reported from India [10] where lower daily dose of 200 mg Voriconazole is effective against *Scedosporium* keratitis. However, in that report, topical Itraconazole 1% was used in contrast to our patient where topical Amphotericin B and topical Voriconazole 1% were used.

Topical Voriconazole 1% as monotherapy is reported to be effective for treatment of *Scedosporium* keratitis with mild anterior chamber inflammation [11]. In a case where inflammatory reaction was severe like in our case, the role of systemic antifungal for better penetration is in no doubt. Our patient's hypopyon takes 5 weeks to clear with topical Amphotericin B, topical Voriconazole, and oral Voriconazole. Lewis et al. reported that Amphotericin B and Voriconazole exhibited similar in vitro pharmacodynamic characteristic against *Scedosporium* species [12]. Both demonstrated the same degree of hyphael damage. However, there was no in vitro study done to assess synergistic effect of these two antifungal drugs. We believe that we are the first to report the successfulness of this combination of antifungal for the treatment of refractory *Scedosporium* keratitis. Unfortunately, we are unable to demonstrate in vitro susceptibility of our isolate to any antifungal. Unlike bacterial culture, susceptibility of fungal culture is not routinely done in our hospital.

In a situation where there is a rapid worsening of the keratitis, an ophthalmologist would use many possible empirical strategies to hedge pharmacokinetics and pharmacodynamic uncertainties. Decision on modalities of treatment will heavily base on clinical response. In all cases of microbial keratitis, tight control of diabetes mellitus and compliancy are important to ensure the success of the treatment. Our case demonstrated that *Scedosporium apiospermum* keratitis is an opportunistic infection, which is difficult to treat despite tight control of diabetes mellitus and intensive antifungal treatment. The infection appeared to have very quick onset but needs long duration of treatment to completely heal. Surgical debridement always plays an important role as a therapeutic procedure as well as establishes the diagnosis through repeat scrapping.
